# Integrating transcriptome and metabolome to explore the growth-promoting mechanisms of GABA in blueberry plantlets

**DOI:** 10.3389/fpls.2023.1319700

**Published:** 2023-12-21

**Authors:** Mingfeng Liu, Mingyue Bai, Jiajia Yue, Xiaoke Fei, Xiuying Xia

**Affiliations:** Plant Cell and Genetic Engineering Laboratory, School of Biological Engineering, Dalian University of Technology, Dalian, China

**Keywords:** blueberry plantlets, gamma-aminobutyric acid, tissue culture, metabolome, transcriptome

## Abstract

Tissue culture technology is the main method for the commercial propagation of blueberry plants, but blueberry plantlets grow slowly and have long growth cycles under *in vitro* propagation, resulting in low propagation efficiency. In addition, the long culturing time can also result in reduced nutrient content in the culture medium, and the accumulation of toxic and harmful substances that can lead to weak growth for the plantlets or browning and vitrification, which ultimately can seriously reduce the quality of the plantlets. Gamma-aminobutyric acid (GABA) is a four-carbon non-protein amino acid that can improve plant resistance to various stresses and promote plant growth, but the effects of its application and mechanism in tissue culture are still unclear. In this study, the effects of GABA on the growth of *in vitro* blueberry plantlets were analyzed following the treatment of the plantlets with GABA. In addition, the GABA-treated plantlets were also subjected to a comparative transcriptomic and metabolomic analysis. The exogenous application of GABA significantly promoted growth and improved the quality of the blueberry plantlets. In total, 2,626 differentially expressed genes (DEGs) and 377 differentially accumulated metabolites (DAMs) were detected by comparison of the control and GABA-treated plantlets. Most of the DEGs and DAMs were involved in carbohydrate metabolism and biosynthesis of secondary metabolites. The comprehensive analysis results indicated that GABA may promote the growth of blueberry plantlets by promoting carbon metabolism and nitrogen assimilation, as well as increasing the accumulation of secondary metabolites such as flavonoids, steroids and terpenes.

## 1 Introduction

Blueberry (*Vaccinium corymbosum* L.) is a small berry fruit tree belonging to the genus *Vaccinium*. Blueberry fruit is rich in anthocyanins and has high nutritional value and healthcare function (Han et al., 2021; Guo et al., 2022). In recent years, the demand for blueberry fruit in the international market has been on the rise, and this has resulted in constant expansion of the blueberry planting area and a substantial increase in the demand for high-quality nursery stock. Tissue culture technology provides an economical and effective method for the large-scale production of pathogen-free and genetically homogeneous blueberry plantlets (Debnath and Goyali, 2020). However, in the implementation of rapid *in vitro* propagation of blueberry plantlets, there are some problems at the production level, such as low growth rate, low reproduction efficiency and long growth cycle, and these problems can impede the rapid renewal of new varieties of blueberry and the rapid development of the blueberry industry. Developing strategies to improve the propagation efficiency and quality of *in vitro* blueberry plantlets is of great significance to the commercial production of blueberry nursery stock and the development of the blueberry industry as a whole.

Although the culture conditions can be artificially regulated, the extreme environmental conditions that may appear in the process of tissue culture, such as high humidity, high concentration of plant regulators, inefficient gas exchange, inappropriate osmotic potential and pH, accumulation of toxic and harmful substances may exert stress on the explants, resulting in physiological and metabolic disorders, growth and development obstacles, and decreased proliferation efficiency (Bakir et al., 2016; Muneer et al., 2016). Improving the ability of the explants to adapt to and resist the adverse conditions of the tissue culture is likely to be beneficial to the growth and reproduction of the plants. A large number of studies have confirmed that exogenous application of melatonin, non-protein amino acids (e.g., gamma-aminobutyric acid, beta-aminobutyric acid and L-3,4-dihydroxyphenylalanine), salicylic acid (SA), jasmonic acid (JA) and other substances can alleviate the damage caused by abiotic stress such as high temperature, drought, salinity, and heavy metals to promote plant growth and development (Ruan et al., 2019; Nadarajah et al., 2021; Wang et al., 2021; Chen et al., 2022; Yu et al., 2022; Chmur and Bajguz, 2023). This provides a novel approach to addressing the challenging conditions frequently encountered in blueberry tissue culture.

Gamma-aminobutyric acid (GABA) is a four-carbon non-protein amino acid, which is a natural product of plants (Podlesakova et al., 2019; Bown and Shelp, 2020). In recent years, GABA has become a new hotspot in the field of plant stress research, and its promoting effects on plant growth and plant stress resistance have been widely reported (Gramazio et al., 2020; Shelp et al., 2021). Studies have confirmed that plants not only use GABA as a metabolite, but also as a signaling molecule (Fait et al., 2008; Rocha et al., 2010; Che-Othman et al., 2020), and that GABA plays an important role in integrating stress signals, regulating gene expression, coordinating a series of physiological and biochemical reactions, and controlling plants stress responses (Shi et al., 2010; Ramesh et al., 2015; Gilliham and Tyerman, 2016; Ansari et al., 2021). A large number of studies have shown that when plants are subjected to stresses (both abiotic and biotic) such as hypoxia, drought, cold, heat, salt, ultraviolet, heavy metals, mechanical damage, and diseases and pests, GABA can rapidly accumulate to maintain the equilibrium of redox, cytoplasmic pH, osmotic potential, and C/N content in the cells to ensure their normal physiological metabolism under adverse conditions and alleviate the extent of damage caused by these stresses (Seifikalhor et al., 2019; Li et al., 2020; Abd Elbar et al., 2021; Mishra et al., 2023; Ullah et al., 2023). The exogenous application of GABA can improve the stress resistance of plants and promote their growth under stress by increasing stomatal conductance, improving the activities of antioxidant enzymes, promoting the production of stress-response amino acids and polyamines, and regulating the metabolism of organic acids among others (Jin et al., 2019; Wang et al., 2022; Ahmadi et al., 2023). Although numerous studies have identified the contribution of GABA to plant growth and development under different stress conditions and the underlying mechanisms of GABA actions, the effects of exogenous GABA on the growth of the *in vitro* plantlets under the extreme artificial conditions of tissue culture and the related molecular mechanisms remain unclear.

Transcriptomics can investigate gene transcription and transcriptional regulation in cells from a global perspective, revealing expression changes across samples and delving into molecular processes. Metabolites, influenced by internal and external factors, reflect plant physiology and can regulate gene transcription. Acting as a genomic-phenotypic bridge, metabolomics unveils plant responses to external stimuli. The comprehensive analysis of metabolomics and transcriptomics can provide more information at the physiological and molecular levels for a deeper understanding of GABA shunt pathway and its impact mechanisms on plant growth.

In this study, the effects of GABA on the growth of *in vitro* blueberry plantlets were studied. Furthermore, changes in the levels of metabolites and gene expression induced by GABA in the plantlets were systematically analyzed by metabonomic and transcriptomic techniques to clarify the mechanisms of GABA. The study aimed to provide technical guidance for improving the propagation efficiency and quality of blueberry plantlets and to provide theoretical support for expanding the application of GABA.

## 2 Materials and methods

### 2.1 Culture and GABA treatment of *in vitro* blueberry plantlets

The *in vitro* plantlets of blueberry variety ‘O’ Neal’ (*Vaccinium corymbosum* L.) used in the experiment were obtained and maintained by School of Bioengineering, Dalian University of Technology through tissue culture method (Gao et al., 2018), and the original branches used for tissue culture were obtained from Dalian LanYuan Technology Development Co., LTD.

Subcultured blueberry plantlets were cut into 1.5 cm segments and inoculated into a proliferation medium supplemented with 0 or 0.5 g·L^-1^ GABA. The proliferation medium consisted of 1/2 MS (Murashige and Skoog, 1962) basic Medium (Qingdao Hope Bio-Technology Co., Ltd.), supplemented with 5.5 g·L^-1^ AGAR, 1 mg·L^-1^ ZT, 20 g·L^-1^ sucrose, pH 5.0 ± 0.1. Each bottle was inoculated with 10 segments of the cut plantlets. The plantlets were cultured under a light intensity of 30-40 µmol·m^-2^·s^-1^, a light duration of 16 h·d^-1,^ and a temperature of (25 ± 2)°C. After 60 days, the height, fresh weight, and multiplication ratio of the plantlets were determined. Ten bottles of plantlets were taken from each treatment for transcriptome and metabolome analysis, with two bottles of plantlets contributing to each biological replicate.

### 2.2 Metabolome profiling

Five biological replicates from each treatment were subjected to metabolic analysis. First, the callus at the base of each plantlet was removed, and the collected plantlets were then put into a lyophilizer, then grinding (60 Hz, 2 min) the samples to powder form by using a grinder. A sample powder (80 mg) of each replicate was transferred into a 2 mL microcentrifuge tube followed by the addition of 20 μL internal standard (L-2-chlorophenylalanine, 0.3 mg/mL; Lyso PC17:0, 0.01 mg/mL) and 1 mL of 70% methanol. The mixture was subjected to ultrasonication for 30 min, kept at -20°C for 20 min, and then centrifuged for 10 min (18,000×*g*, 4°C). An aliquot (300 μL) of the supernatant was dried in a vacuum centrifuge. The sample was then re-dissolved in 400 μL of 25% methanol, vortexed repeatedly for 30 s, exposed to ultrasonication for 3 min, and then kept at -20°C for 2 h. After that, the mixture was centrifuged at 18,000×*g* at 4°C for 10 min and the supernatant was subjected to UPLC-MS/MS analysis.

The metabolites were separated by chromatography using an ACQUITY UPLC (Waters, USA) equipped with an ACQUITY UPLC BEH C18 column (100×2.1 mm, 1.7 µm; Waters, Milford, USA). A 2 μL sample was loaded onto the column, which was then eluted with a mobile phase consisting of 0.1% formic acid in water (A) and 0.1% formic acid in a mixture of acetonitrile and methanol (2:3 v/v) (B). The column was operated at 45°C and under a flow rate of 0.4 mL/min. The eluent was further analyzed by a high-resolution tandem mass spectrometer AB Triple TOF 5600 plus (AB Sciex, USA) to identify the metabolites eluted from the column. The ESI source operation parameters were as follows: source temperature 500°C; ion spray voltage (IS) 5500 V (positive ion mode)/-4500 V (negative ion mode); ion source gas I (GSI), gas II (GSII), curtain gas (CUR) were set at 50, 60, and 35 psi, respectively; the collision-activated dissociation (CAD) was high. DP (declustering potential) and CE (collision energy) for individual MRM transitions was done with further DP and CE optimization. A specific set of MRM transitions were monitored for each period according to the metabolites eluted within this period.

For data acquisition, the UNIFI 1.8.1. software was used. The obtained scaled data set was imported into Progenesis QI v2.3 (Nonlinear Dynamics, Newcastle, UK) for PCA, PLS discriminant analysis (PLS-DA), and orthogonal partial least squares discriminant analysis (OPLS-DA) in order to observe the maximum metabolic changes of each group at all-time points. Differentially accumulated metabolites (DAMs) were defined as those metabolites having a *P*-value < 0.05 (obtained from a two-tailed Student’s t-test) and VIP > 1 (obtained from the OPLS-DA model).

### 2.3 Transcriptome profiling

Total RNA was extracted from blueberry plantlets using the Total RNA Extractor (Trizol) kit (Sangon Biotech Shanghai, China). A total amount of 1 µg RNA per sample was used as input material for the RNA sample preparations. A TruSeq Stranded mRNA Library Preparation Kit (Illumina) was employed to generate cDNA libraries, following the standard protocol. Briefly, mRNAs in the total RNA were isolated by Oligo (dT) magnetic beads and used to synthesize the cDNA. The RNA sample was subsequently fragmented into sizes ranging from 200 to 600 bp for a duration of 6 minutes under the influence of divalent cations at 85°C. Both the first- and second-strand cDNA synthesis procedures were conducted using the cleaved RNA fragments. Hieff NGS™ DNA Selection Beads were used to purify the cDNA. Subsequently, the cDNA fragments underwent ligation with indexed adapters, A-tailing, and end-repair processes. Then PCR was performed with Phusion High-Fidelity DNA polymerase, Universal PCR primers and Index (X) Primer. At last, PCR products were purified (Hieff NGS™ DNA Selection Beads, 0.9×, Beads : DNA=1:1) and library quality was assessed on the Agilent 2200 TapeStation (Agilent Technologies, Germany). RNA sequencing was performed on a MGISEQ-2000 sequencing system (BGI Genomics, China).

Use FastQC (v0.11.2) and Trimmomatic (v0.36) to control and filter the original data. All subsequent analyses are based on clean reads. Transcriptome assembly was performed using Trinity (v2.40). Use TransDecoder (v3.0.1) to identify candidate coding regions within transcript sequences generated by *de novo* RNA-Seq transcript assembly using Trinity.

Gene expression levels were calculated by Salmon (v0.8.2). Differences in gene expression levels were analyzed using DESeq2 (v1.12.4) with the filter set to *P*-value < 0.05, and the difference between multiples (FoldChange) > 2. TopGO (v2.24.0) was used for GO enrichment analysis and clusterProfiler (v3.0.5) was used for KEGG pathway analysis.

### 2.4 Statistical analysis

All experimental data were presented as means ± standard errors. SPSS 21.0 software was used for T-test and Duncan’s shortest significant ranges, and GraphPad Prism 5.0 was used for plotting.

## 3 Results

### 3.1 Effects of GABA on the growth of *in vitro* blueberry plantlets

**Figure 1:**
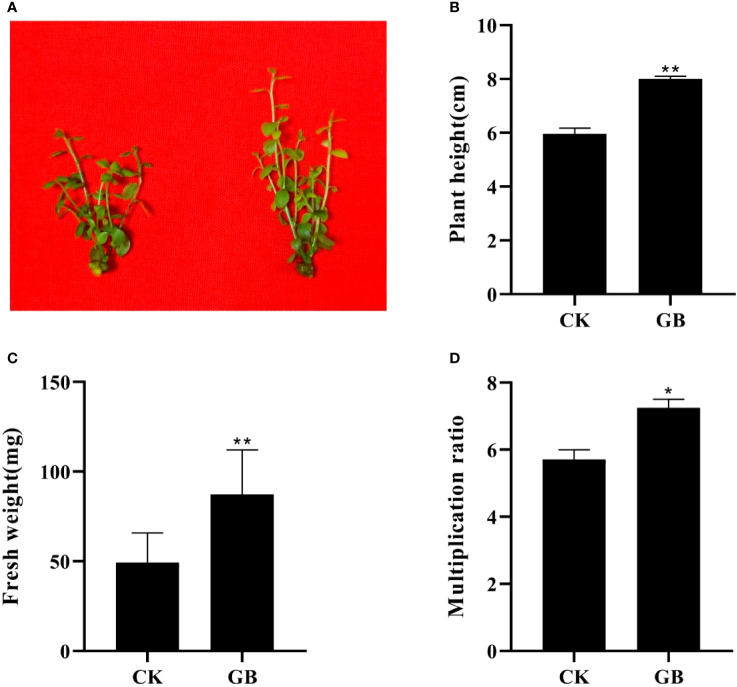
Effects of GABA on the morphology and growth in *Vaccinium* plantlets *in vitro.* **(A)** Morphology characteristics; **(B)** plant height; **(C)** fresh weight; **(D)** multiplication ratio. CK, control; GB, 0.5 g·L^-1^ GABA. Data are shown as the mean ± SEM. Significance of control vs 0.5 g·L^-1^ GABA is indicated by one (*P* < 0.05) or two (*P* < 0.01) asterisks.

Compared with the control plantlets (no GABA treatment), the plantlets treated with GABA showed obvious morphological changes manifested as enlarged leaves and thickened stems (**Figure 1A**). The height, fresh weight, and proliferation multiple of GABA-treated plantlets increased significantly, and by as much as 34.08%, 77.04%, and 27.01%, respectively, compared with the control plantlets (**Figures 1B–D**). The exogenous application of GABA could, therefore, promote growth and improve the quality of cultured blueberry plantlets.

### 3.2 Multivariate statistical analysis of metabolomics and screening of differential metabolites.

**Figure 2:**
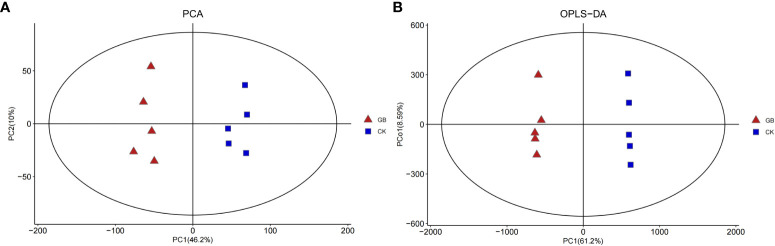
PCA **(A)** and OPLS-DA **(B)** analysis diagram of metabolites in *Vaccinium* plantlets *in vitro* samples.

A total of 4,376 metabolites were detected in the blueberry plantlets. Principal component analysis (PCA) (**Figure 2A**) showed that the interpretation rates of the two principal components, PC1 and PC2 were 46.2% and 10%, respectively. All samples of the GABA-treated group (GB) were located on the negative axis, while all samples of the control group (CK) were located on the positive axis, indicating obvious separation. Furthermore, the results of the orthogonal partial least squares-discriminant analysis (OPLS-DA) and 200-response sorting tests (**Supplementary Figure 1**) indicated that the model was stable and reliable, allowing for a follow-up study to be conducted (**Figure 2B**).

According to OPLS-DA data and Student’s t-test, 377 differential metabolites with VIP > 1 and a *P*-value < 0.05 were selected from the 4,376 metabolites. Among them, 187 metabolites were up-regulated and 190 were down-regulated by GABA treatment (**Figure 2A**). The classification of the differential metabolites showed the greatest changes for lipids and lipid-like molecules (42.7%), followed by phenylpropanoids and polyketides (12.73%), organic acids and derivatives (7.16%), and organooxygen compounds (6.63%) (**Supplementary Figure 2**). The results indicated that GABA exerted an important impact on the metabolisms of lipids, phenylpropanoids, and organic acids in blueberry plantlets.

**Figure 3:**
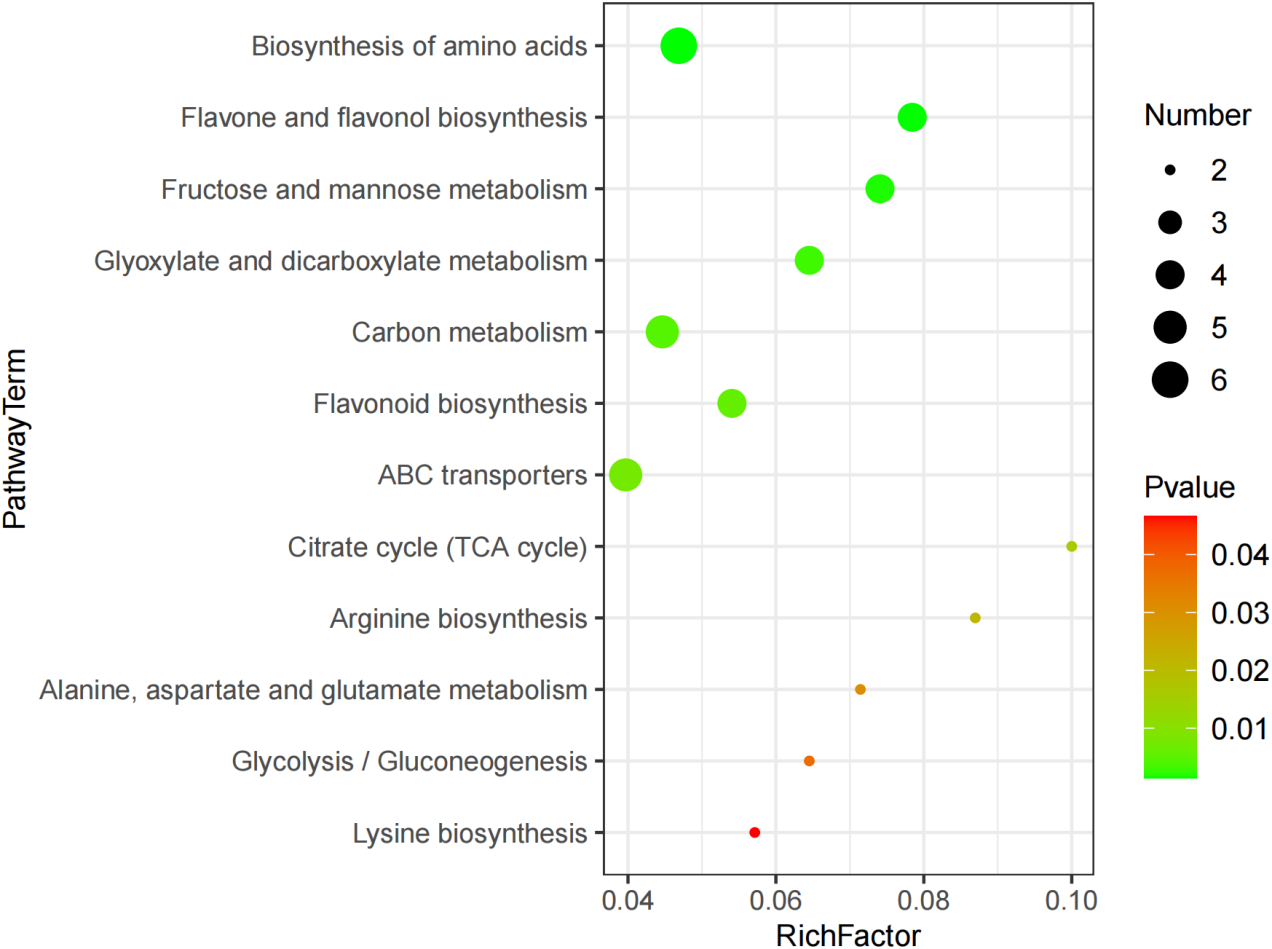
Bubble diagram of differential metabolic pathway.

The KEGG enrichment analysis revealed 12 metabolic pathways being significantly affected by GABA, including the biosynthesis of amino acid, ABC transporter, flavone, flavonol, and flavonoid and the metabolism of carbon, fructose, mannose, glyoxylate, and dicarboxylate (**Figure 3**). These metabolic pathways mainly involved 27 DEMs, including 4 amino acids and organic acids such as lysine, arginine, and citric acid, 4 carbohydrates such as α-D-glucose, pyro1-phosphoric acid, and arbutin, and 4 phenolic acids such as 1-caffeylquinic acid and epicatechin. The result suggested that these metabolites might play a key role in the GABA-mediated regulation of blueberry growth.

### 3.3 Screening and KEGG enrichment analysis of differentially expressed genes (DEGs)

**Figure 4:**
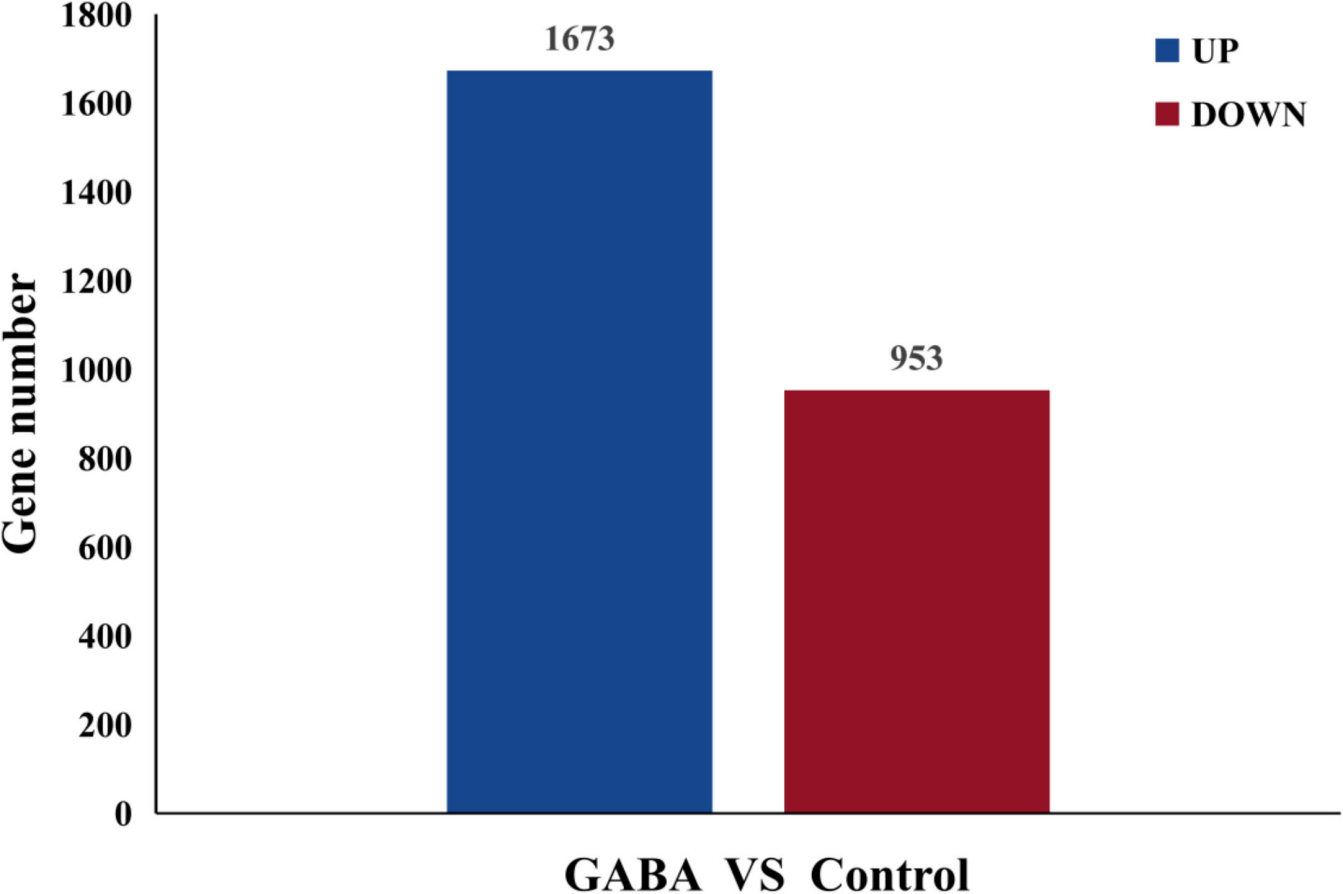
Differential expressed genes histogram.

RNA sequencing results obtained for the GABA-treated and control plantlets were used for metabolomics analysis. A total of 2,626 genes were differentially expressed in blueberry plantlets following GABA treatment, with 1,673 genes being upregulated and 953 genes being down regulated compared with the plantlets that did not receive GABA treatment (**Figure 4**).

**Figure 5:**
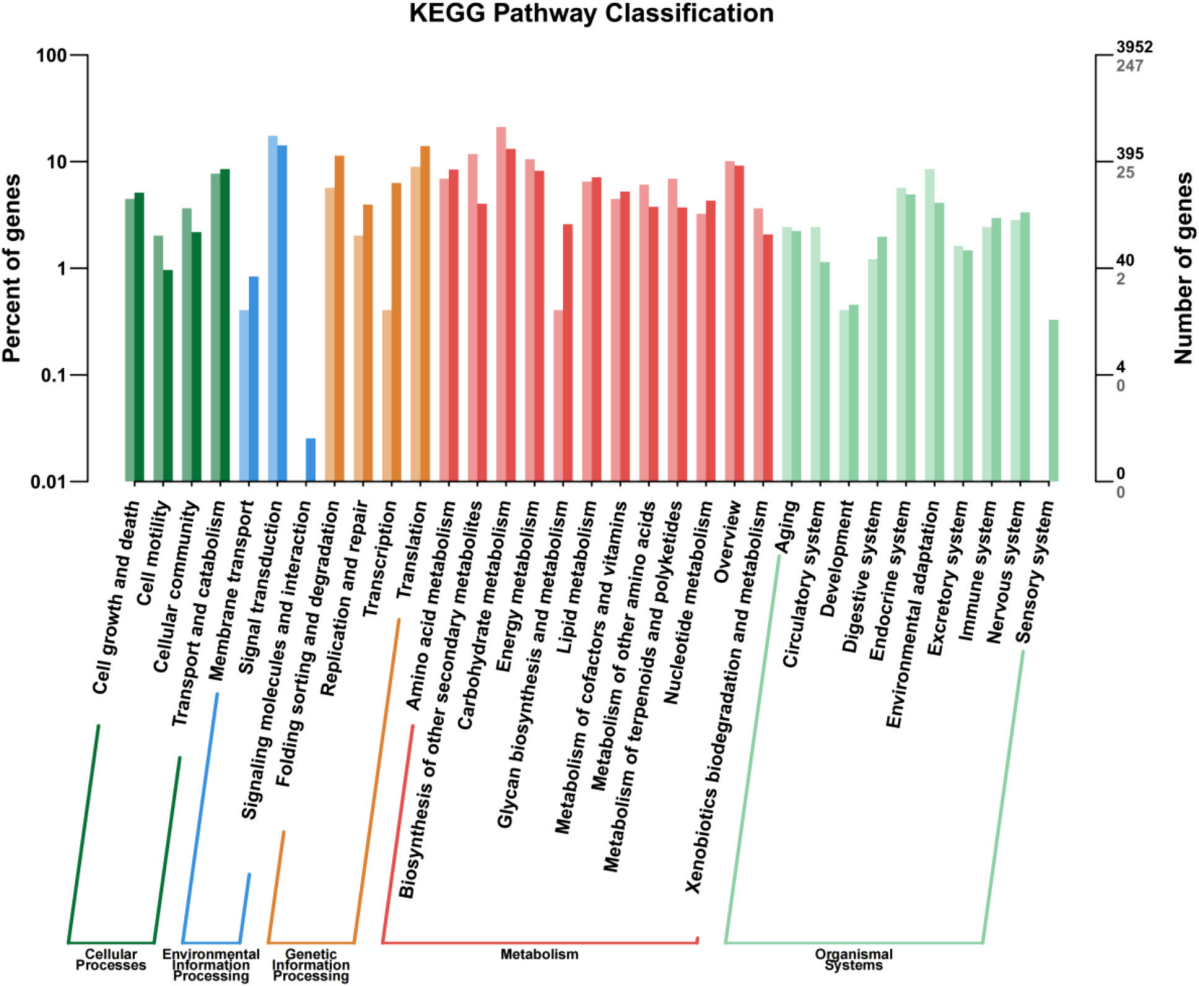
KEGG pathway categories of differentially expressed genes.

Annotation of the DEGs by the KEGG pathway placed 2,626 DEGs in 188 pathways, which were further divided into five categories: cellular process, environmental information processing, genetic information processing, metabolism, and organismal systems, among which, metabolism and organismal systems were the most enriched categories (**Figure 5**). In the cell process category, most DEGs were associated with transport, catabolism, cell growth, and cell death pathways. In the environmental information processing category, the highest proportion of DEGs was associated with the signal transduction pathway, followed by amino acid metabolism, lipid metabolism, energy metabolism, and biosynthesis of other secondary metabolites. In the genetic information processing category, the translation pathway accounted for the highest proportion of DEGs. In the organismal systems, the endocrine system and environmental adaptation were the two main pathways found to associate with most of the DEGs. These metabolic pathways may play an important role in the GABA-promoted growth of *in vitro* blueberry plantlets.

Notably, both the transcriptome and metabolome revealed enrichment of carbohydrate metabolism and biosynthesis of secondary metabolites, suggesting that GABA significantly affected the physiological processes of carbon and nitrogen metabolism and the secondary metabolism of blueberry plantlets.

### 3.4 Effect of GABA on carbon and nitrogen metabolism of plantlets

**Figure 6:**
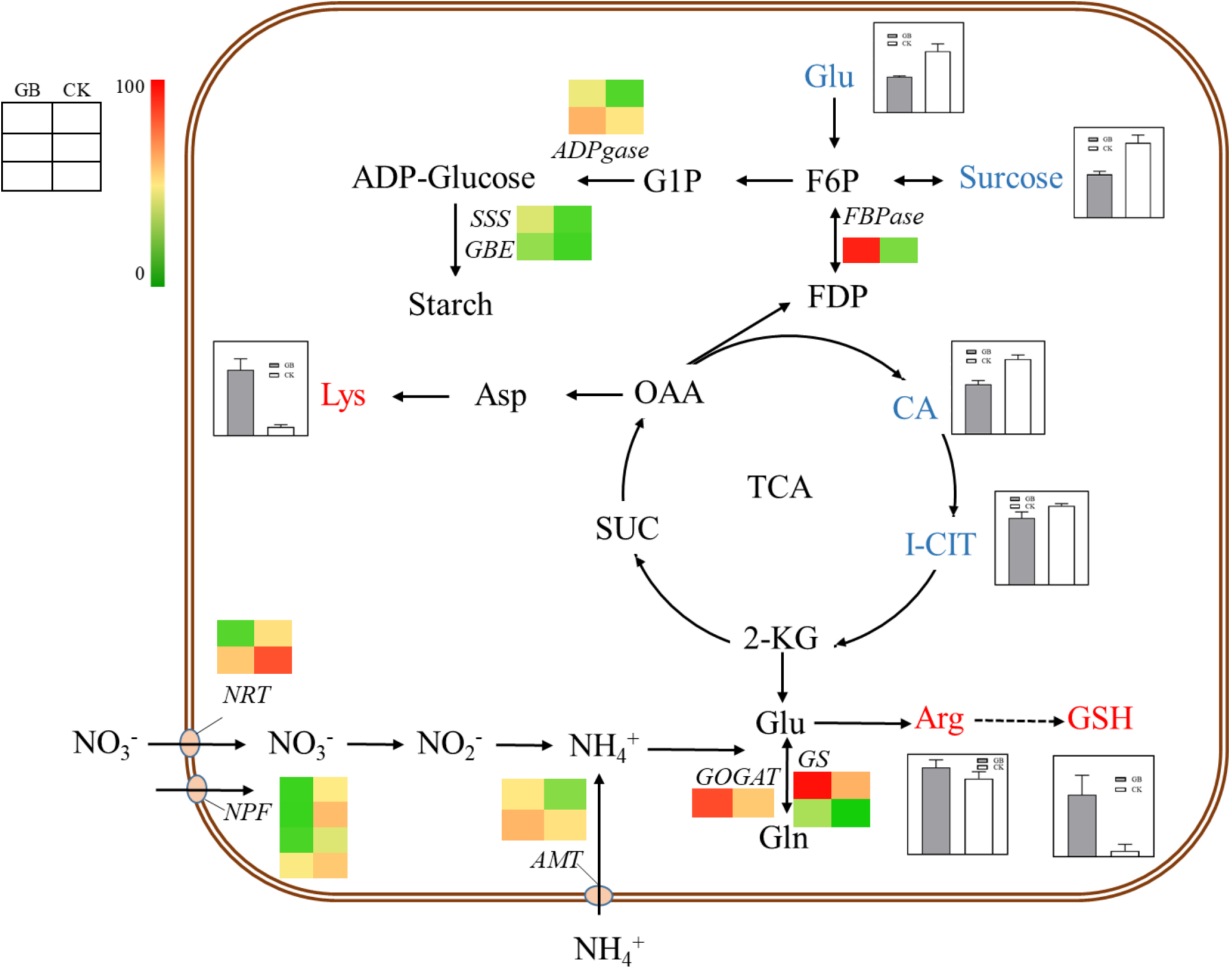
Carbon and nitrogen metabolism map. The histogram in the diagram indicates the change of metabolite content, and the heat map indicates the change of gene expression. *NRT*, *Nitrate Transporter*; *NPF*, *NRT/PTR*; *AMT*, *Aminomethyl Transferase*; *GOGAT*, *Glutamate Synthase*; *GS*, *Glutamine Synthetase*; *FBPase*, *Fructose 1, 6-bisphosptase*; *ADPGase*, *ADP-glucose Pyrophosphorylase*; *SSS*, *Soluble Starch Synthase*; *GBE*, *1,4-alpha-glucan-branching Enzyme*; Glu, Glutamate; Gln, Glutamine; Arg, Arginine; GSH, Glutathion; CA, Citric Acid; I-CIT, Isocitric acid; KGA, α-Ketoglutaric acid; SUC, Succinic acid; OAA, Oxalacetic acid; Asp, Aspartic acid; Lys, Lysine; FDP, Fructose1,6-diphosphate; F6P, Fructose 6 phosphate; G1P, Glucose 1-phosphate.

Transcriptome and metabolome analysis showed that many DEGs and DEMs were enriched in the carbon and nitrogen metabolic pathways. Specifically, the contents of sucrose, glucose, citric acid, and isocitrate were significantly decreased, while the contents of arginine, lysine, and glutathione were significantly increased in the GABA-treated plantlets (**Figure 6**). Accordingly, the expression levels of genes related to nitrogen assimilation, and starch and cellulose synthesis, including *GOGAT* (glutamate synthetase), *GS* (glutamine synthetase), *ADPgase* (ADPG pyrophosphorylases), *SSS* (Soluble starch synthases), *GBE* (glucan-branching enzyme) and *CesA* (cellulose synthase), were significantly increased in the GABA-treated plantlets. In addition, the expression level of the fructose 1,6-bisphosphatase gene (*FBPase*), a key gene in the gluconeogenesis pathway, was also significantly up-regulated in the GABA-treated plantlets. It is worth noting that GABA treatment also significantly upregulated the expression of the ammonium transporter gene *AMT*, while significantly down-regulated the expression of two nitrate transporter genes, *NRT* and *NPF* in these plantlets.

Based on the combined results of metabolome and transcriptome, we proposed that GABA might have a harmonizing effect on the carbon and nitrogen metabolism of blueberry plantlets, and this could provide sufficient raw materials and energy for the synthesis of starch, cellulose, and protein by promoting the absorption of ammonium nitrogen and assimilation of nitrogen. Such effects would thereby, enhance sucrose degradation, glycolysis, tricarboxylic acid cycle, and gluconeogenesis, eventually resulting in the promotion of growth and development for the plantlets. GABA, therefore, might play an important role in regulating the absorption and transport of different forms of nitrogen sources in blueberry plantlets.

### 3.5 Effects of GABA on the secondary metabolism of blueberry plantlets

**Figure 7:**
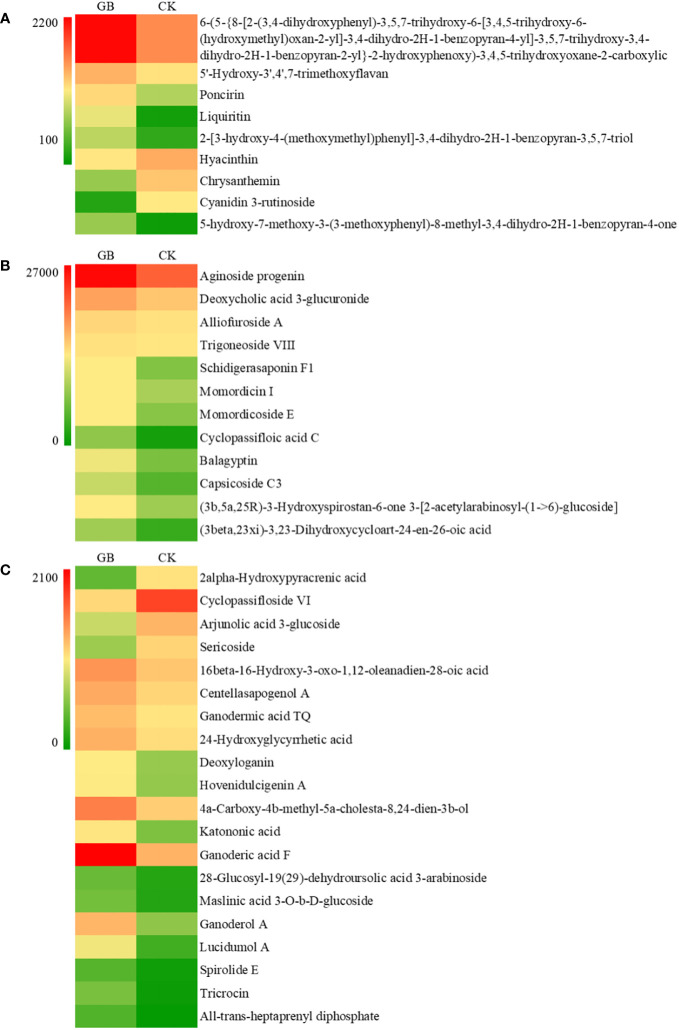
Effects of GABA on the contents of flavonoids **(A)**, steroids and steroid derivatives **(B)** and prenol lipids **(C)** in *Vaccinium* plantlets *in vitro*.

The contents of many secondary metabolites in the GABA-treated plantlets changed significantly compared with the control plantlets. The secondary metabolites included flavonoids, steroids, and their derivatives, as well as isopentenol lipids. A total of 41 different metabolites were selected according to their VIP values (VIP>1), *P*-values (*P* < 0.05) and fold change (|FC|>2) (**Figure 7**). The contents of 5 flavonoids and isoflavones (including poncirin and liquiritin), 12 steroids and their derivatives (including aginoside progenin and deoxycholic acid 3-glucuronide), and 16 isopentenol lipids (including ganoderic acid and deoxyloganin) were significantly increased in the GABA-treated plantlets. At the same time, the expression levels of many key genes involved in the synthesis of terpenes, especially carotenoids, were significantly upregulated (**Table 1**). Such genes included the triterpene synthase, terpene synthase, *PDS* (phytoene desaturase), *CRTISO* (carotenoid isomerase), *LUT2* (lycopene ϵ-cyclase protein), *HYB* (beta-carotene hydroxylase 2), *VDE1* (violaxanthin de-epoxidase), *NCED1* (9-cis-epoxycarotenoid dioxygenase) and *CYP* (cytochrome P450 CYP72A219) genes. The result suggested that GABA may play an important role in regulating the synthesis of flavonoids and carotenoids. GABA may increase the synthesis of secondary metabolites such as flavonoids and terpenes to improve the antioxidant and anti-stress ability of blueberry plantlets to maintain growth of the plantlets under an adverse environment.

**Table 1:** Differential genes related to terpene synthesis.

Gene ID	Gene name	Expression level of 0.5 g·L^−1^ GABA group	Expression level of 0 g·L^−1^ GABA group	pValue	log_2_Fold Change
TRINITY_DN29690_c0_g1	*triterpene synthase*	44.52	15.50	7.14E-06	1.52
TRINITY_DN28561_c2_g2	*terpene synthase*	22.13	5.33	6.49E-07	2.00
TRINITY_DN22806_c2_g1	*terpene synthase*	52.05	17.90	1.43E-04	1.54
TRINITY_DN22806_c2_g2	*terpene synthase*	41.08	13.45	2.79E-05	1.61

## 4 Discussion

Tissue culture technology is the main method for the commercial production of blueberry plantlets. However, under *in vitro* propagation, blueberry plantlets grow slowly, with a culture cycle of more than 60 days, so it is difficult to reproduce the required quantity in a short time, and this has limited the rapid availability of new varieties. In addition, with the extension of culture time, the nutrient content in the culture medium will decrease and toxic substances might start to accumulate, resulting in the aging, browning, and vitrification of the plantlets, and this can seriously reduce the quality and the survival rate of the plantlets following transplanting. How to improve the proliferation efficiency and quality of the blueberry plantlets under *in vitro* propagation has been an urgent problem that needed to be solved in the commercial production of blueberry plantlets. A large number of studies have confirmed that the exogenous application of GABA can effectively alleviate the damage to plants caused by drought, salinity, high temperature, heavy metals, and other stresses while promoting plant growth and improving plant yield and quality (Jin et al., 2019; Liu et al., 2021; Xu et al., 2021; Shan et al., 2022; Li et al., 2022b; Mishra et al., 2023; Yuan et al., 2023). Our data also confirmed that the application of exogenous GABA under tissue culture conditions could promote the growth and differentiation of blueberry plantlets, improve their quality and can be applied to the commercial production of blueberry seedlings. This has been the first time that GABA was used in the tissue culture field, and it could expand the scope of GABA application in agriculture. We posit that GABA can serve as a fundamental component in the culture medium for the rapid propagation of blueberries or other plants. Nevertheless, further experiments are essential to explore the impact of GABA on the growth of different plant species and to assess the potential adverse effects arising from its continuous application.

Carbon metabolism and nitrogen metabolism are the most basic material and energy acquisition processes in plants and therefore, these processes have an important effect on the growth, development, and formation of yield and quality of the plants. GABA can regulate carbon and nitrogen metabolism and carbon and nitrogen balance by providing the substrate succinic acid for the TCA cycle (Michaeli and Fromm, 2015). Barbosa et al. (Barbosa et al., 2010) found that exogenous GABA can as a signaling molecule, modulating the activities of enzymes involved in primary nitrogen metabolism and nitrate uptake. Du et al. (Du et al., 2020) have demonstrated that GABA in chestnut seeds negatively regulates the development of the adventitious root by altering carbon and nitrogen metabolism. We have shown that exogenous GABA significantly reduced the contents of intermediates products in glycolysis and the TCA cycle, such as sucrose, glucose, citric acid, and isocitrate, and significantly increased the contents of lysine, arginine, and glutathione in blueberry plantlets. The key genes of gluconeogenesis (*FBPase*), starch synthesis (*ADPgase*, *SSS*, *GBE*), and cellulose synthesis (*CesA*) were significantly up-regulated in the GABA-treated plantlets. These results suggested that GABA could balance carbon and nitrogen metabolism in blueberry plantlets under tissue culture conditions, consistent with previous studies performed on other plant species under field or greenhouse conditions. According to our analysis, GABA treatment might provide sufficient energy and raw materials for the synthesis of polysaccharides and proteins by promoting glycolysis, the TCA cycle and gluconeogenesis while promoting the synthesis and accumulation of starch and cellulose by enhancing the expression of related genes, thereby promoting the growth of the plantlets.

It has been proven that the activity of nitrite reductase (NR) in blueberries is low, so it is generally believed that blueberry plants prefer to absorb ammoniacal nitrogen (Alt et al., 2017). The results of the transcriptome analysis that we obtained revealed a significant increase in the expression of the *AMT* gene and a significant decrease in the expression of the *NRT* and *NPF* genes in blueberry plantlets after treatment with GABA, imply that GABA may promote the absorption of ammonia nitrogen and reduce the absorption of nitrate nitrogen. However, the specific effects of GABA on nitrogen uptake by plants need further experimental verification. Exogenous application of GABA under Ca(NO_3_)_2_ stress has been shown to enhance the activities of NR, glutamine synthetase (GS), and glutamine oxoglutarate aminotransferase (GOGAT) in melon seedlings, and improve the absorption of NO_3_^–^N and the assimilation of NH_4_^+^-N in melon roots and leaves under stress conditions (Zhen et al., 2018). At the same time, by limiting glutamate dehydrogenase (GDH) to inhibit the release of NH_4_^+^-N, the ammonia poisoning caused by melon seedlings under stress conditions is alleviated (Li et al., 2022a). GS and GOGAT are the key enzymes of nitrogen assimilation. These enzymes can convert the ammonia nitrogen and nitrate nitrogen absorbed by plants into amino acids to meet the needs of the plants for nitrogen and also to alleviate the toxicity to the plants caused by a high concentration of free ammonia nitrogen (Zhen et al., 2018). It was clear that the expression of the *GS* and *GOGAT* genes in blueberry plantlets was upregulated by the exogenous GABA (**Figure 6**). GABA probably regulated nitrogen metabolism and promoted the growth in blueberry plantlets by enhancing the ability of the plantlets to absorb different nitrogen sources and improve their nitrogen assimilation ability. Thus, GABA could promote the growth of *in vitro* blueberry plantlets by regulating the absorption of different forms of nitrogen fertilizers.

The secondary metabolites in plants are mainly divided into terpene (terpenoids, steroids), phenols (phenylpropanoids, quinones, flavonoids, tannins), and nitrogenous compounds (alkaloids), and these all play an important role in antioxidant capacity, stress resistance, maintenance of homeostasis and growth (Sohn et al., 2022). Phenols can eliminate reactive oxygen species (ROS) and are important antioxidant components in plants. Terpenes play an important role in plant biofilm construction, auxin synthesis, pigment synthesis, and photosynthesis (Alwakil et al., 2022). Studies on barley and soybean have shown that exogenous GABA can promote the expression of related genes, increase the content of phenolic substances in seedlings, and improve the antioxidant capacity and adaptability of the plants in response to adverse conditions (Zhu et al., 2019; Zhao et al., 2021). With *in vitro* blueberry plantlets, many DEGs and DEMs that were enriched following treatment with GABA were associated with the biosynthetic pathways of flavonoid and carotenoid. In addition, these plantlets also displayed significant increases in both the expression of genes related to terpene biosynthesis and the actual levels of most terpenes (terpenes, steroids) and flavonoids. These results provided evidence that the application of exogenous GABA to *in vitro* blueberry plantlets could increase the content of flavonoids, terpenes, steroids, and other secondary metabolites in the plantlets, improve the adaptability of the plantlets to their environments, and maintain the growth of the plantlets in the late stage of tissue culture.

## 5 Conclusion

**Figure 8:**
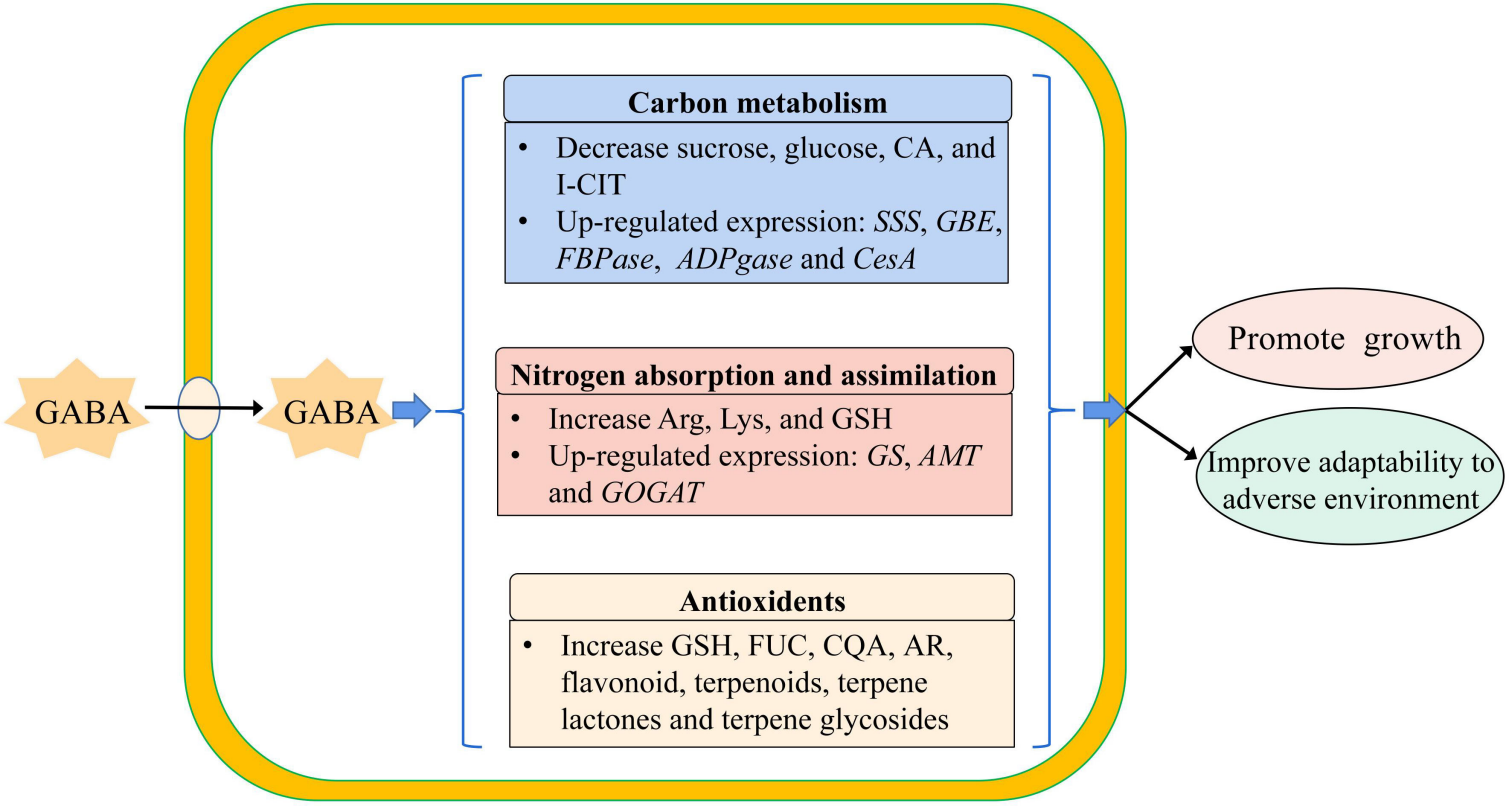
The model of exogenous GABA improving the adaptability of plantlets *in vitro* to the adverse environment. SSS, Soluble Starch Synthase; GBE, 1,4-alpha-glucan-branching Enzyme; FBPase, Fructose 1, 6-bisphosptase; ADPGase, ADP-glucose Pyrophosphorylase; CesA, cellulose synthase; GS, Glutamine Synthetase; AMT, Aminomethyl Transferase; GOGAT, Glutamate Synthase; CA, Citric Acid; I-CIT, Isocitric acid; Arg, Arginine; Lys, Lysine; GSH, Glutathion; FUC, Fucose; CQA, Caffeoylquinic acid; AR, Arbutin.

Based on the result obtained from the combined transcriptome and metabolome analysis of GABA-treated *in vitro* blueberry plantlets versus untreated plantlets, the growth-promoting effect of GABA could be attributed to two main mechanisms. Firstly, GABA-promoted carbon metabolism and nitrogen absorption, processes that could provide more energy and substrates for cellular activities such as the synthesis of starch, cellulose, and protein, resulting in an increased dry matter. Secondly, GABA enhanced the adaptability of the plantlets to adverse environments via enhanced regulation of secondary metabolism to increase the production of secondary metabolites such as flavonoids, steroids, and terpenes, which could maintain the growth of the plantlets in the later stage of the culture (**Figure 8**).

## Data availability statement

The datasets presented in this study can be found in online repositories. The names of the repository/repositories and accession number(s) can be found below: https://www.ncbi.nlm.nih.gov/, PRJNA976102; https://www.ncbi.nlm.nih.gov/, PRJNA977259.

## Author contributions

ML: Writing – original draft. MB: Investigation, Writing – original draft. JY: Software, Writing – original draft. XF: Data curation, Writing – original draft. XX: Writing – review & editing, Writing – original draft.
