# Supplementary material for: Integrating transcriptome and metabolome to explore the growth-promoting mechanisms of GABA in blueberry plantlets
Source: Front Plant Sci. 2023 Dec 21;14:1319700. doi: 10.3389/fpls.2023.1319700 (PMC10768180; doi:10.3389/fpls.2023.1319700)
Supplement: Supplementary file 1 [file DataSheet_1.docx]

Supplementary Material

# Supplementary Figures

#
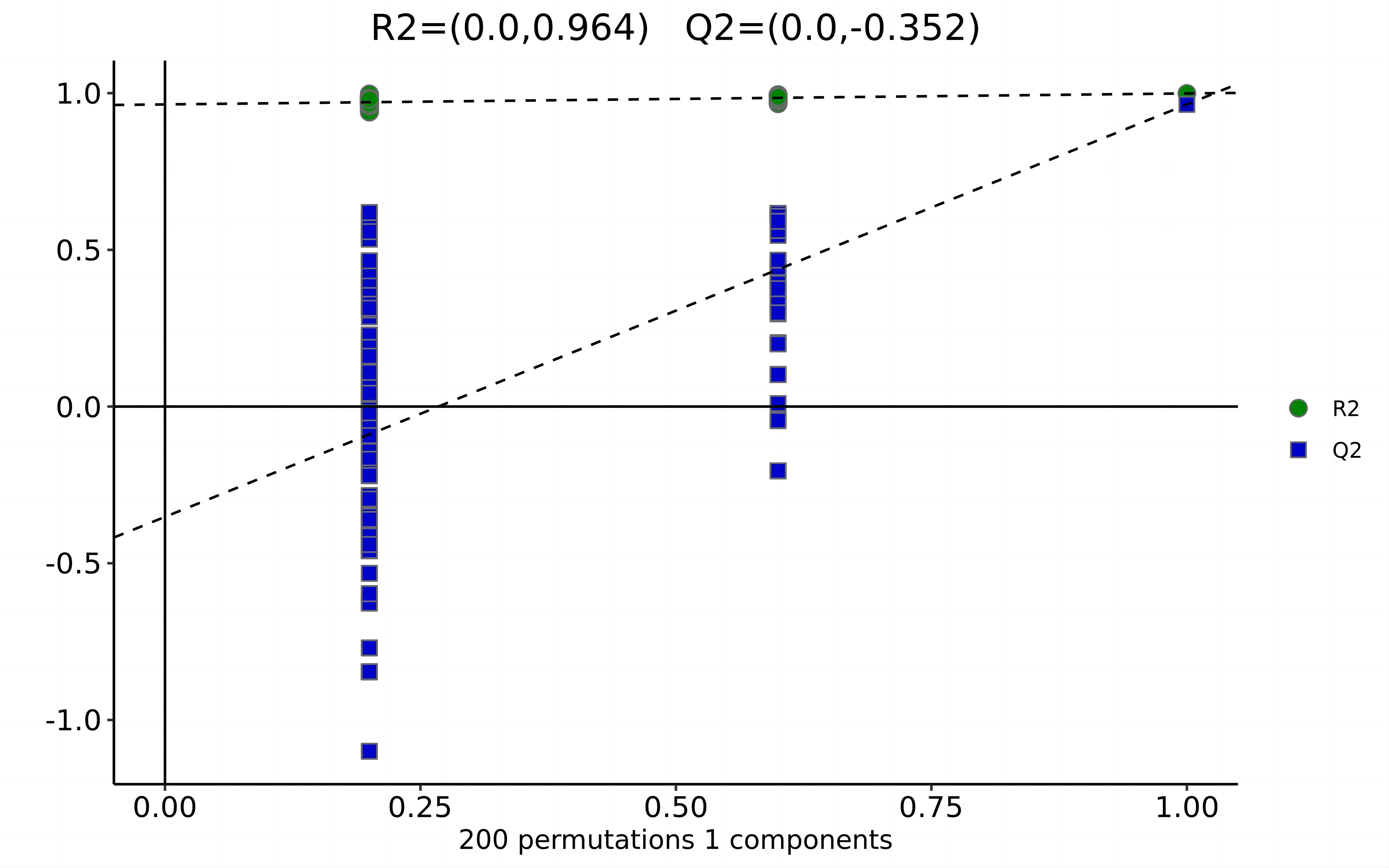


# Supplementary Figure 1. Response sequencing test to verify whether the model is over-fitting.


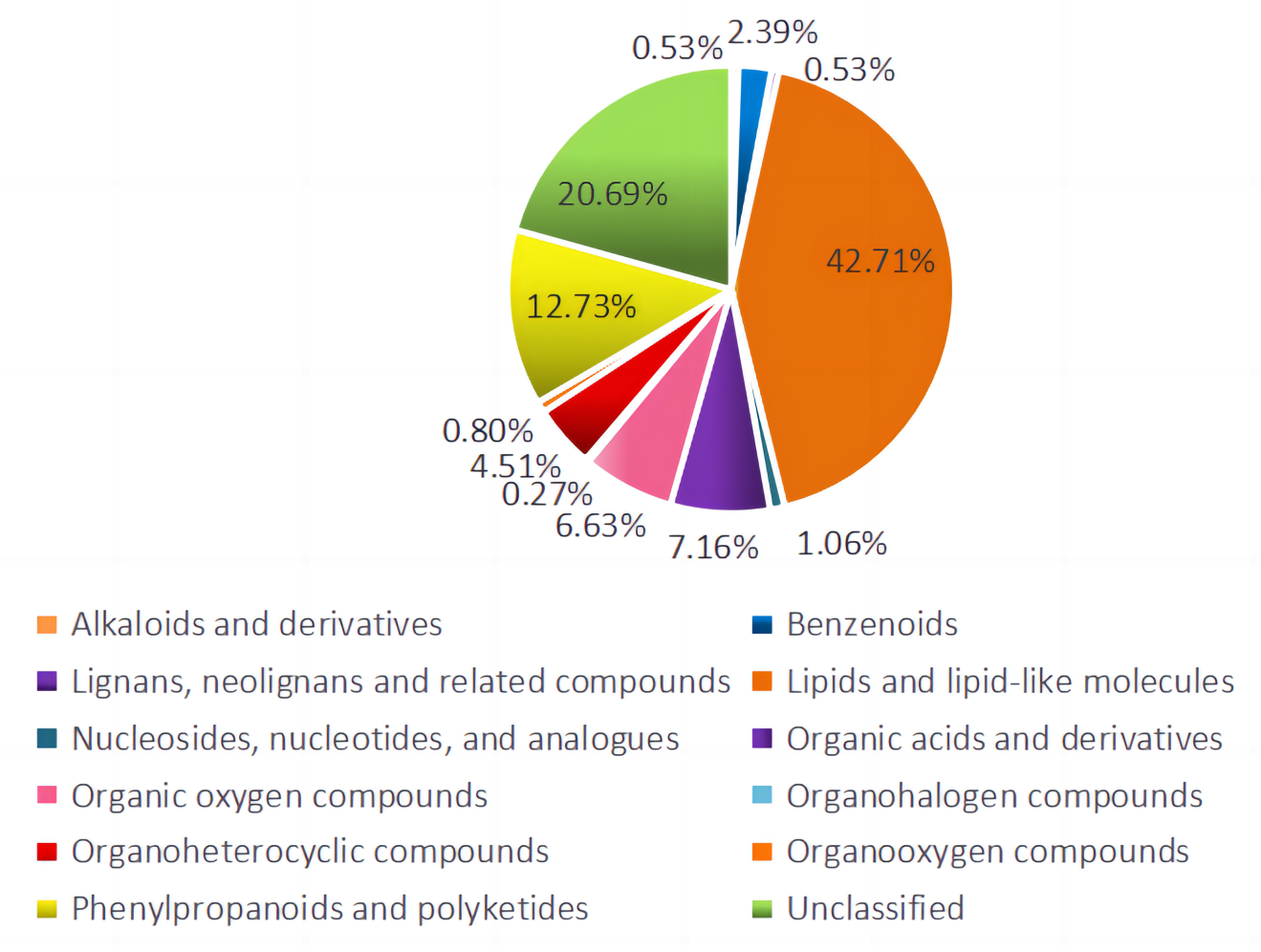


**Supplementary Figure 2.** Differential metabolite classification map.
